# GC-MS Analysis of Volatile Differences in Rice and Qingke Noodles Formulated with Functional Root Plant Flours

**DOI:** 10.3390/molecules31081348

**Published:** 2026-04-20

**Authors:** Essam ElShamey, Jiazhen Yang, Jiachun Jiang, Xiaoying Pu, Li Xia, Li’e Yang, Xiaomeng Yang, Yawen Zeng

**Affiliations:** 1 Biotechnology and Germplasm Resources Institute, Yunnan Academy of Agricultural Sciences, Kunming 650205, China; 2 Rice Research Department, Field Crops Research Institute, Agricultural Research Center, Giza 33717, Egypt; 3 Tea and Coffee College of Pu’er University, Pu’er 665000, China

**Keywords:** GABA, rice noodles, qingke noodles, GC-MS, metabolic, functional foods

## Abstract

The integration of rooted plant flour into traditional noodle matrices, such as rice noodles and qingke noodles, represents a novel approach to enhancing the nutritional and sensory profiles of staple foods. This study investigates the volatile flavor components and functional compounds derived from rooted plant flours, including Gongmi “tribute rice”, qingke “highland barley” flour, kudzu vine flour, *Gastrodia elata* blume flour, dried ginger flour, and fishwort root flour, when incorporated into rice and qingke noodles. The novelty of this research lies in its comprehensive analysis of how these flours influence not only the nutritional and textural properties but also the volatile organic compounds (VOCs) that define sensory acceptance and health benefits. Using advanced gas chromatography mass spectrometry (GC-MS), we identified key VOCs, such as esters, aldehydes, and terpenes, which contribute to unique flavor profiles like umami, sweetness, and earthy notes in fortified noodles. Additionally, the study highlights the best functional compounds for health, including polyphenols, resistant starch, and polysaccharides, which demonstrate significant antioxidants, anti-inflammatory, and cholesterol-lowering properties. For instance, highland barley enriched flour exhibited high levels of phenolic compounds and carotenoids, which correlated with improved antioxidant activity and a reduced glycemic index. Similarly, Gongmi flour contributed elevated levels of γ-aminobutyric acid (GABA) and rutin, enhancing the rice noodles’ potential to manage metabolic diseases and support cardiovascular health. Molecular docking analyses predicted strong interactions between key volatile compounds (e.g., 3-dihydro-1, 3-trimethyl-33-phenyl-1H-indene) and metabolic targets like ACE and SGLT1, suggesting mechanisms for their cardioprotective and anti-diabetic effects. This research provides a groundbreaking framework for developing next generation functional foods by leveraging rooted plant flours to bridge the gap between sensory appeal and health efficacy, offering strategic insights for personalized nutrition and sustainable food production.

## 1. Introduction

The growing demand for functional foods has driven research into incorporating alternative flour sources into traditional staple foods like noodles. Among these, rooted plant flours derived from tubers, roots, and other underground plant parts have gained significant attention for their nutritional benefits and unique flavor profiles [1]. These flours, obtained from sources such as Gongmi, highland barley, kudzu root, *Gastrodia elata* blume, ginger root, and various grains, contribute not only enhanced nutritional value but also distinct aromatic characteristics that significantly influence the overall sensory experience of noodle products. The study of volatile flavor compounds in these ingredients has become crucial for developing palatable functional foods that meet consumer expectations while providing health benefits beyond basic nutrition [2]. The incorporation of rooted plant flours into noodle matrices represents an innovative approach to enhancing both the nutritional and sensory properties of traditional foods [3]. Rice noodles, a staple in many Asian cuisines, and qingke noodles, derived from highland barley and representing Tibetan culinary traditions, provide excellent bases for such functional enhancements [4]. Understanding the volatile flavor components and their behavior during processing is essential for optimizing product formulation and processing techniques to create functional noodles with superior sensory attributes and consumer appeal. This paper aims to synthesize current research on the volatile flavor profiles of various rooted plant flours and their impact on the sensory characteristics of rice and qingke noodles, providing insights for future development of functional noodle products.

Gongmi, often referred to as “tribute rice,” represents a category of historically significant cereal grains renowned for their exceptional quality and cultural importance in Chinese history [5]. Gongmi, historically an imperial grain prized for its superior sensory and nutritional qualities, has garnered renewed scientific and commercial interest in the modern era. Valued for its nutritional density, unique flavor, and cultural significance, Gongmi represents a promising resource for functional food innovation. Its extensive genetic diversity, a product of centuries of cultivation, holds potential for discovering novel health-beneficial compounds. Yet, this potential is jeopardized by contemporary challenges such as genetic stagnation and climate susceptibility. This introduction outlines the journey of Gongmi from ancient prestige to modern relevance, framing its study as crucial for leveraging historical agro-biodiversity to address current food security and nutritional needs [6,7]. Certain traditional Gongmi rice varieties now face extinction, unable to thrive within modern mechanized agriculture. This threat underscores a pressing scientific imperative: to systematically characterize the unique volatile and functional compounds within these historic grains. Such research is foundational for effective conservation and sustainable cultivation. Furthermore, deciphering this chemical profile is essential for accurate quality assessment, reliable origin tracing, and the creation of value-added products. Ultimately, this scientific understanding is vital not only for preserving agricultural biodiversity but also for promoting these culturally significant grains for future generations. Highland barley, known as qingke in Mandarin, has served as a dietary cornerstone for local populations for centuries [8,9]. Unlike common barley varieties, highland barley thrives in the extreme conditions of high altitudes, intense UV radiation, and low oxygen levels, which contribute to its unique phytochemical composition [10]. Recent years have witnessed growing scientific interest in this cereal due to its rich content of bioactive compounds and potential health benefits, including hypoglycaemic effects, cholesterol-lowering properties, and antioxidant activities [11]. The flour derived from highland barley contains diverse volatile organic compounds that determine its aroma characteristics, as well as numerous functional components that contribute to its nutritional value and health promoting effects [12,13]. The sensory and nutritional profiles of highland barley products are fundamentally shaped by their processing, which alters key volatile and functional chemical components. Despite its recognized nutritional value, this cereal remains underutilized in global food production, largely owing to limited knowledge of its specific chemical composition and transformation during processing. This study provides a comprehensive analysis of the structural properties, nutritional significance, and processing-induced changes in the volatile and functional compounds of highland barley flour, aiming to bridge this critical knowledge gap.

The growing demand for functional foods has spurred innovation in traditional staples, such as rice and qingke noodles. A promising strategy involves incorporating flours from rooted plants, which can significantly enhance both the sensory appeal and nutritional profiles of these products. These flours contribute complex aromas, derived from volatile compounds like aldehydes and ketones, that can distinguish new functional offerings in the marketplace. However, successful integration requires optimizing processing parameters to preserve these desirable aromas and functional properties. This research investigates the use of rooted plant flours as value-added ingredients, which offers a dual potential: to create nutritious, flavorful noodles that align with consumer trends and to leverage economically advantageous crops for local agricultural development. The main target of this research is to develop and optimize innovative noodle products (specifically rice and qingke noodles) by incorporating rooted plant flours. This core objective breaks down into several key aims: To utilize the unique volatile compound profiles (aldehydes, ketones, alcohols) of rooted plant flours to create distinct and desirable aroma characteristics in noodles. To enhance the nutritional or health-beneficial qualities of the noodles through the addition of these plant-based ingredients. To identify and carefully manage the critical processing parameters that influence the retention and transformation of volatile aroma compounds during noodle production. To demonstrate the commercial viability of these innovative products by meeting consumer demand for functional foods while highlighting the economic benefits of using locally sourced, value-added rooted plant flours.

## 2. Results

### 2.1. Volatile Compounds in Rooted Plant Flours

Rooted plant flours exhibit diverse and complex volatile compound profiles that contribute significantly to their distinctive aroma characteristics [14]. Research on tuberous roots such as *Gastrodia elata* blume, dried ginger, and fishwort root has revealed that these materials contain substantial levels of amino acids, carotenoids and polyphenols, which not only provide nutritional benefits but also influence the flavor profiles through their degradation products [15]. When incorporated into extruded snacks, these root flours significantly enhanced color saturation and texture crispness, suggesting similar potential for noodle applications. Noodles (rice and qingke) contain many components, whether pure or added in proportion to other plant components. During this research, we will discuss each component separately and the added value of the final product (noodles).

#### 2.1.1. The Volatile and Functional Profile of Root of Kudzu Flour

**Figure 1:**
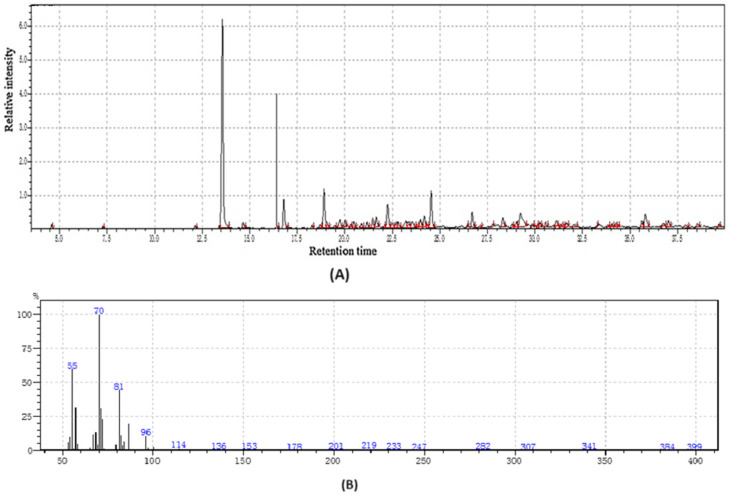
GC-MS two-way data in chromatography for root of kudzu vine flour (**A**,**B**).

Kudzu root flour represents a nutritionally complex ingredient whose value is defined by two distinct yet complementary groups of compounds; volatile organic compounds and functional bioactive compounds as shown in Table 1. The volatile compounds are primarily responsible for the flour’s unique sensory profile. These aromatic molecules, including aldehydes, alcohols, and terpenes, impart the characteristic earthy, sweet, and slightly nutty aroma that influences the acceptability and application of kudzu flour in culinary contexts as shown in Figure 1. Beyond flavor, the volatile profile acts as a key indicator of the flour’s quality, processing conditions, and shelf-life stability, as changes in these compounds can signal oxidation or spoilage.

However, the true functional supremacy of kudzu root flour lies in its rich concentration of bioactive compounds, most notably the isoflavones like puerarin and daidzein. These compounds are not merely nutritional, they are pharmacologically active, underpinning the flour’s traditional medicinal uses and modern health appeal [16,17]. They confer potent antioxidant, anti-inflammatory, and potential cardioprotective and neuroprotective benefits. The presence of starch with unique functional properties, along with dietary fiber and other micronutrients, further enhances its value as a functional food, contributing to digestive health and potentially modulating blood glucose levels [18]. Ultimately, the interplay between these two groups of compounds defines kudzu flour’s identity.

#### 2.1.2. The Volatile and Functional Profile of Fishwort Root Flour

Fishwort root flours represent a promising and undervalued source of nutrition and bioactive compounds with their value derived from two distinct yet important classes of chemicals, volatile compounds and functional non-volatile compounds [19], as shown in Table 1. The volatile compounds, including terpenes like taraxasterol and phenylpropanoids, are primarily responsible for the flour’s distinct aromatic profile, contributing earthy, green, and slightly bitter notes. While these volatiles influence sensory acceptance, they are also potent bioactive agents, contributing to the plant’s traditional medicinal uses through anti-inflammatory and antioxidant activities.

**Figure 2:**
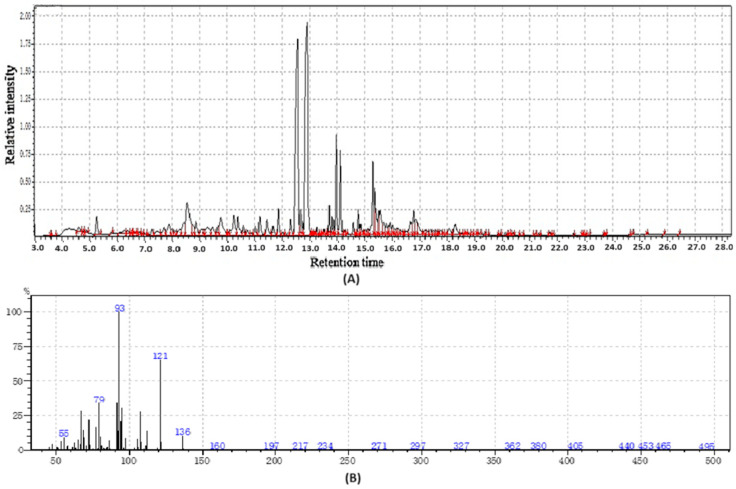
GC-MS two-way data in chromatography for fishwort root flour (**A**,**B**).

Fishwort root flour functions as a significant functional food ingredient due to its high concentration of non-volatile compounds as shown in Figure 2, including phenolic acids and flavonoids with potent antioxidant activity, alongside prebiotic fibers like inulin and essential minerals. These constituents are collectively associated with demonstrated health benefits such as improved gut health, enhanced blood sugar regulation, and better mineral absorption, which, combined with its volatile aroma profile, establishes its value and supports its potential for broader application in the functional food industry [20].

#### 2.1.3. The Volatile and Functional Profile of *Gastrodia elata* Blume Flour

**Figure 3:**
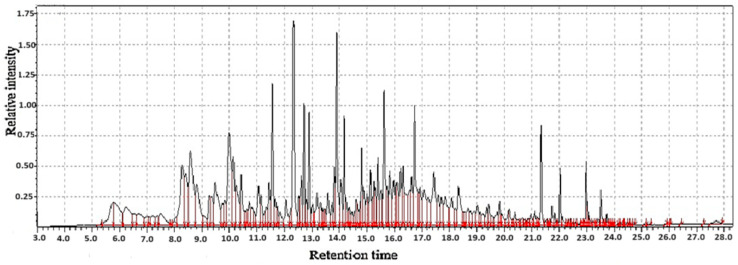
GC-MS two-way data in chromatography for *Gastrodia elata* flour.

The analysis of *Gastrodia elata* blume flours reveals a complex and synergistic profile of volatile and functional (non-volatile) compounds that underpin its dual identity as both a valued traditional medicine and a promising functional food ingredient as shown in Table 1. The volatile organic compounds (VOCs), primarily consisting of phenolic aldehydes (like 4-hydroxybenzaldehyde), alcohols, acids, and esters, are responsible for the flour’s distinctive earthy, herbal, and slightly sweet aroma as shown in Figure 3. The phenylpropanoids, most notably gastrodin and parishins, are the principal bioactive constituents, acclaimed for their potent neuroprotective, sedative, anticonvulsant, and memory-enhancing properties. These are complemented by a rich portfolio of other compounds, including polysaccharides that modulate the immune system and promote gut health, beta-sitosterol with its anti-inflammatory benefits, and a suite of antioxidants that mitigate oxidative stress. It represents a concentrated source of bioactive agents with significant potential for developing nutraceuticals, functional foods, and dietary supplements aimed at promoting neurological health, reducing inflammation, and combating oxidative stress [21].

#### 2.1.4. The Volatile and Functional Profile of *Polygonatum sibiricum* Redoute Flour

**Figure 4:**
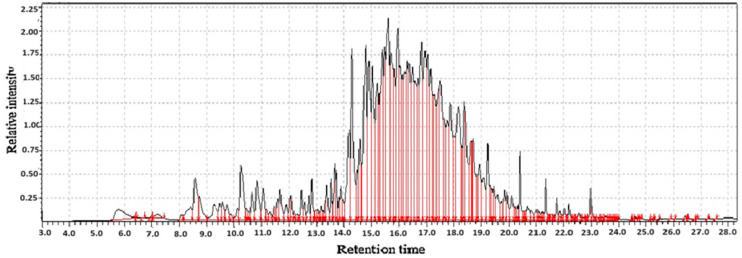
GC-MS two-way data in chromatography for *Polygonatum sibiricum* Redoute flour.

The analysis of *Polygonatum sibiricum* Redoute flours reveals a complex and sophisticated phytochemical profile that underpins its dual identity as both a functional food and a revered medicinal ingredient. The distinct character of the flour is derived from the intricate interplay between its volatile aromatic compounds and its non-volatile, bioactive functional constituents. The volatile organic compounds (VOCs) are responsible for the flour’s unique sensory identity. The presence of compounds such as hexanal, nonanal, benzaldehyde, and various furans and pyrazines imparts a complex aroma profile characterized by green, grassy, sweet, and roasted notes as shown in Table 1 and Figure 4. This aroma is not merely a sensory attribute but can also serve as a key indicator of quality, processing methods (particularly drying and heating), and potential authenticity. The abundance of polysaccharides, saponins (e.g., steroidal saponins like kingianoside), flavonoids, and phenolic acids directly contributes to its documented antioxidant, immunomodulatory, anti-fatigue, anti-aging, and hypoglycemic activities [22].

#### 2.1.5. The Volatile and Functional Profile of Dried Ginger Flour

**Figure 5:**
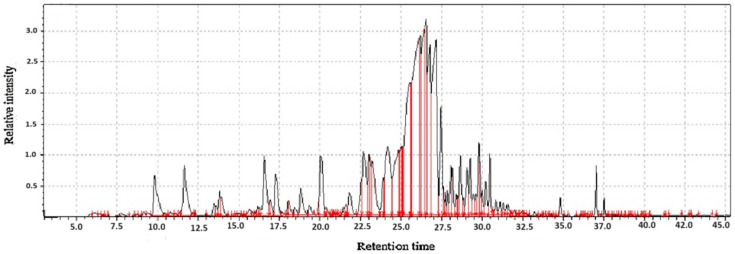
GC-MS two-way data in chromatography for dried ginger flour.

Dried ginger flour represents a concentrated and versatile form of ginger, whose unique value is fundamentally defined by its two distinct yet interconnected classes of chemical constituents: volatile compounds and functional (non-volatile) compounds. The volatile compounds, primarily terpenes like zingiberene, arcurcumene, and *β*-sesquiphellandrene as shown in Figure 5, are responsible for the characteristic aroma and flavor profile of ginger as shown in Table 1. However, the drying process significantly alters this volatile profile. While some fresh, bright top notes are inevitably lost due to their high volatility, the process often concentrates other compounds and can lead to the formation of new, deeper aroma molecules like shogaols. This results in the distinctively warm, spicy, and less pungent aroma that defines dried ginger, setting it apart from its fresh counterpart [23,24]. Conversely, the functional compounds, notably the gingerols and their dehydrated derivatives, the shogaols, are the powerhouse behind ginger’s renowned health-promoting properties. The conversion of gingerols into shogaols during thermal dehydration creates compounds with significantly heightened antioxidant, anti-inflammatory, and potential anticancer activities [25,26]. While this reduces the pungency associated with fresh ginger (gingerols), it amplifies its therapeutic potential [27,28].

The volatile compounds deliver the sensory experience essential for culinary acceptance, while the functional compounds provide the substantiated health benefits that elevate it from a simple spice to a functional food ingredient [29].

### 2.2. Impact of Rice Noodles Volatile Profiles and Quality

Volatile compounds are the aromatic molecules responsible for the distinctive smell and flavor of rice noodles, while primarily made from just rice and water, these noodles develop a complex profile of volatiles through processing and cooking. Key compounds often include aldehydes, alcohols, and hydrocarbons. The specific profile is heavily influenced by the rice variety, fermentation (in certain types like river noodle), and most importantly, heat treatments during drying.

#### 2.2.1. Analysis of Volatile and Functional Compounds in Gongmi Plant Flours

Gongmi represents a category of historically significant cereal grains renowned for their exceptional quality and cultural importance in Chinese history [30], these premium grains were traditionally reserved for imperial consumption due to their superior sensory attributes and perceived nutritional benefits [16]. The study of Gongmi plant flour extends beyond their historical value to encompass their potential contributions to modern functional food development and nutritional security [31].

The comprehensive analysis of volatile and functional compounds in Gongmi plant flours relies on advanced analytical technologies that enable precise identification and quantification of complex chemical constituents. GC-MS provides enhanced capabilities for identifying and quantifying a broader range of volatile and non-volatile compounds as shown in Table 2, including those present in trace amounts.

**Figure 6:**
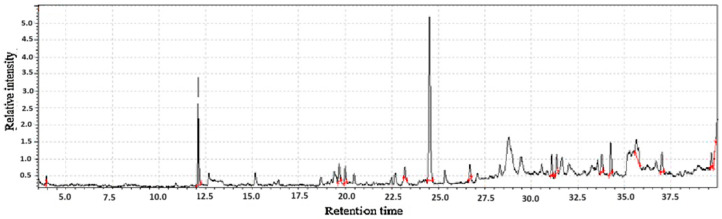
GC-MS two-way data in chromatography for Gongmi plant flours.

This technique enables the detection and identification of volatile compounds at very low concentrations, providing a comprehensive overview of the aroma-active compounds that contribute to Gongmi’s distinctive sensory properties. Comprehensive studies using GC-MS technology have identified 74 distinct peaks in Gongmi flours as shown in Figure 6, corresponding to 69 known volatile compounds and five unknown substances, with aldehyde compounds predominating across all varieties, followed by alcohols and esters. These volatile compounds originate from various biochemical pathways including lipid oxidation, amino acid degradation, and glycoside hydrolysis, and their relative abundances vary significantly among different Gongmi varieties based on genetic factors, environmental conditions, and post-harvest processing methods. These volatile compounds not only contribute to the sensory appeal of Gongmi flours but also serve as chemical markers for authenticity testing.

Beyond their volatile compounds, Gongmi plant flours contain a diverse array of bioactive constituents that contribute significant health benefits and functional properties to food products [32]. The non-volatile functional compounds in Gongmi flours include nutritionally valuable components such as peptides, dietary fibers, phenolic compounds, and minerals, which offer various health benefits including antioxidants, antihypertensive, and mineral-binding properties.

#### 2.2.2. The Volatile and Functional Profile of (90% Gongmi Flour + 10% Kudzu Root Flour)

**Figure 7:**
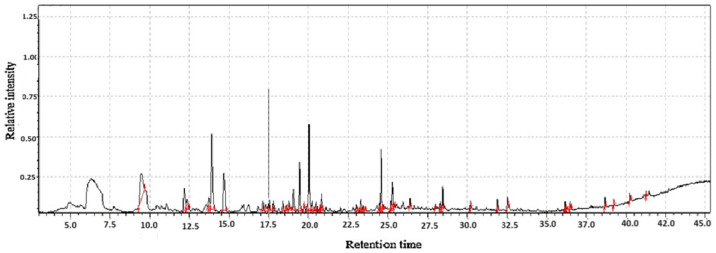
GC-MS two-way data in chromatography for (90% Gongmi flour + 10% kudzu root flour).

The integration of 90% Gongmi flour with 10% kudzu root flour represents far more than a simple ingredient substitution; it is a strategic and synergistic formulation that fundamentally enhances both the aromatic identity and functional performance of the base flour. The analysis of its volatile and functional profile reveals a composite material with significant potential for innovative food applications, bridging the gap between traditional staples and modern nutritional demands. The most immediate impact of incorporating kudzu powder is the dramatic shift in the volatile compound profile. Gongmi flour, while a superb functional base, is often characterized by a relatively neutral and mild aroma as shown in Table 2. Key volatile compounds from kudzu such as aldehydes (imparting green, grassy notes), terpenes (contributing earthy, pine-like undertones), and potentially certain esters (fruity nuances) interlace with the subtle, starchy notes of rice flour as shown in Figure 7. This creates a new, multi-layered aromatic signature that is more robust and complex. It moves the product away from a “blank canvas” towards an ingredient with its own desirable, earthy character, which can reduce the need for artificial flavor enhancers in final products and appeal to consumers seeking “wholefood” sensory experiences.

The kudzu powder, with its gel forming and hydrocolloid-like properties, acts as a powerful binder and structure builder. It effectively mimics some of gluten’s functionality, trapping gases more efficiently during fermentation and yielding final products with improved volume, a more open crumb structure, and reduced structural collapse [33]. Gongmi flour is a source of energy, but kudzu introduces a valuable dietary fiber component, along with its profile of isoflavones (like daidzin) and minerals [34]. This elevates the composite flour from a mere source of calories to a functional food ingredient with potential digestive health benefits and added phytonutrient value, aligning perfectly with contemporary consumer trends towards health and wellness.

#### 2.2.3. The Volatile and Functional Profile of (90% Gongmi Flour + 10% Fishwort Root Flour)

**Figure 8:**
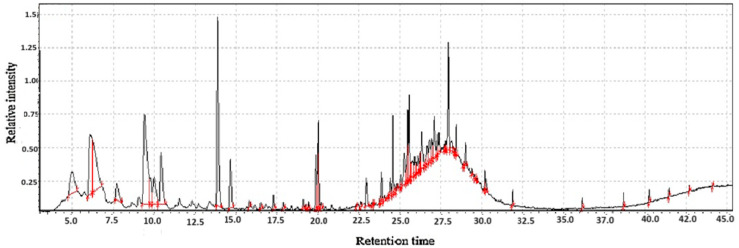
GC-MS two-way data in chromatography for (90% Gongmi flour + 10% fishwort root flour).

The strategic formulation of a composite flour, blending 90% Gongmi flour with 10% fishwort root flour, represents far more than a simple ingredient substitution. The volatile profile of this composite flour is a narrative of transformation, Gongmi flour, while prized for its mild, slightly sweet, and nutty baseline, provides a neutral and accommodating canvas [35]. On its own, it lacks aromatic complexity. The incorporation of 10% fishwort root flour fundamentally alters this landscape, fishwort root flour introduces a powerful and distinctive suite of volatile compounds primarily aldehydes (benzaldehyde) and ketones (tetrapentacontane) as shown in Table 2 and Figure 8. These compounds are responsible for their characteristic citrusy, herbaceous, and slightly “fishy” or pungent aroma. The genius of the 90:10 ratio lies in its balance. The 10% fishwort root flour is sufficient to imbue the blend with a noticeable and intriguing aromatic complexity, elevating it from a simple staple to a gastronomically interesting ingredient. However, it is not so dominant as to be overpowering.

The functional enhancements are where the true promise of this composite flour is realized. Gongmi flour provides the essential structural backbone starch for energy and a modest amount of protein [36]. However, its nutritional profile is relatively conventional. The addition of fishwort root flour acts as a potent functional upgrade; an antioxidant powerhouse is the most significant functional gain [37]. *Houttuynia cordata* is renowned for its high concentration of polyphenols, flavonoids, and other bioactive compounds [38]. These components donate a robust antioxidant capacity to the flour blend, enabling it to scavenge free radicals [39].

#### 2.2.4. The Volatile and Functional Profile of (90% Gongmi Flour + 10% *Gastrodia elata* Blume Flour)

The integration of 10% *Gastrodia elata* blume flour into a Gongmi flour-based matrix (90:10 ratio) represents more than a simple ingredient substitution; it is a strategic, synergistic fusion that fundamentally and favorably alters the product’s volatile and functional profile. Gongmi flour, likely derived from non-glutinous rice, provides a neutral base, good water absorption, and the ability to form cohesive structures [40]. The incorporation of 10% GE (*Gastrodia elata* blume) flour introduces a complex of bioactive compounds as shown in Table 2 and Supplementary Figure S1, primarily gastrodin and parishins, which are the cornerstone of its functional benefits. The GE flour may act as a modifier, potentially lowering the peak viscosity and increasing the stability of the starch gel, resulting in a final product with a more desirable texture less prone to retrogradation and stalling. This leads to improved shelf-life and a more consistent mouthfeel. The most profound functional enhancement is nutritional. The composite flour transitions from a simple source of carbohydrates to a functional food. The presence of GE’s bioactive compounds imbues the final product with potential neuroprotective, antioxidant, and anti-inflammatory properties. This elevates its status from a staple food to a nutraceutical or health-promoting food, aligning with modern consumer demands for foods that support cognitive health and overall well-being.

The true success of the 90:10 blend lies in the principle of synergy. The 10% inclusion level is critical as it is potent enough to impart significant functional and aromatic benefits without overwhelming the system. A higher percentage might lead to an overly strong medicinal flavor or negatively impact the dough’s structural integrity. This synergy makes the composite flour an ideal ingredient for developing innovative food products, such as functional noodles, biscuits, breakfast cereals, or even gluten-free pastries, that cater to the health-conscious consumer without compromising on taste or quality.

#### 2.2.5. The Volatile and Functional Profile of (90% Gongmi Flour + 10% *Polygonatum sibiricum* Redoute Flour)

**Figure 9:**
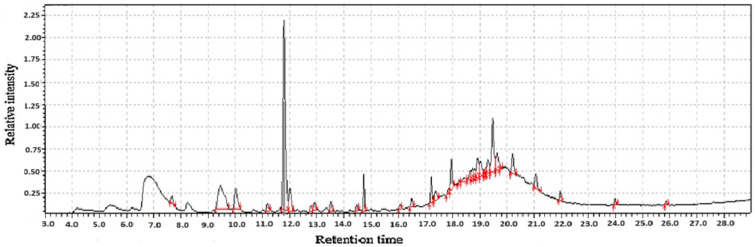
GC-MS two-way data in chromatography for (90% Gongmi flour + 10% *Polygonatum sibiricum* Redoute flour).

The strategic formulation of a composite flour comprising 90% Gongmi flour and 10% *Polygonatum sibiricum* Redouté flour represents more than a simple mixture; it is a sophisticated synergy that yields a novel ingredient with a superior and complex volatile and functional profile. The incorporation of 10% *P. sibiricum* flour acts as a masterful aromatic enhancer, introducing a layer of complexity that elevates the sensory experience far beyond ordinary grain flours. The likely presence of compounds such as hexanal (imparting fresh, green notes), along with various terpenes and aldehydes from the *Polygonatum*, introduces subtle sweet, floral, earthy, and slightly medicinal undertones as shown in Table 2 and Figure 9.

This infusion drastically increases the blend’s antioxidant capacity, shifting its role from a simple source of energy to an active functional ingredient capable of mitigating oxidative stress. The soluble fiber and polysaccharides from *P. sibiricum* significantly alter the flour’s hydration properties. We can anticipate an increase in water and oil absorption capacities, which would enhance dough handling, improve moisture retention in final products (extending shelf-life), and potentially increase yields [41].

#### 2.2.6. The Volatile and Functional Profile of (90% Gongmi Flour + 10% Dried Ginger Flour)

**Figure 10:**
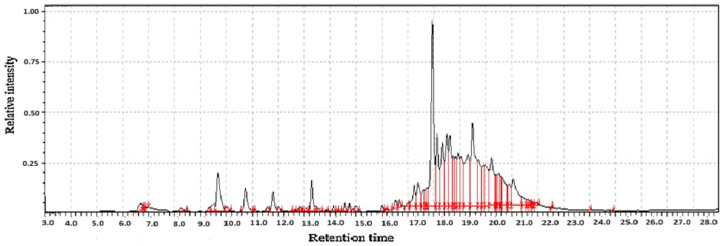
GC-MS two-way data in chromatography for (90% Gongmi flour + 10% dried ginger flour).

The formulation of a composite flour comprising 90% Gongmi flour and 10% dried ginger flour represents far more than a simple mixture of two ingredients; it is a strategic and synergistic fusion that creates a novel food matrix with a significantly enhanced volatile and functional profile as shown in Table 2 and Figure 10. This blend successfully transcends the inherent limitations of Gongmi flour alone, transforming it from a humble, traditional grain base into a sophisticated, multifunctional ingredient poised for modern health conscious and gourmet applications. The most immediate and impactful transformation occurs in the volatile profile. The 10% incorporation of dried ginger acts as a powerful aromatic catalyst, fundamentally reshaping the sensory identity of the flour. These compounds impart a warm, pungent, and slightly citrusy character that is immediately perceptible. Beyond the dominant pungency, the ginger contributes a suite of secondary volatile compounds like bicyclol, caryophyllene, and hexadecane [23]. This adds layers of complexity subtle woody, spicy, and camphorous notes that prevent the aroma from being one dimensional and creates a rich, lingering olfactory experience. This powerful volatile profile effectively masks any potential faint mustiness or grassiness that can be associated with some millet flours, thereby improving overall consumer acceptability. The aroma is not just additive but integrative, suggesting a product that is inherently spicy and warm, rather than a plain flour with an added flavor.

The incorporation of ginger is the primary driver of this shift. The flour blend becomes a significant source of gingerols and shogaols, compounds renowned for their potent antioxidant, anti-inflammatory, and potential anti-nausea and digestive properties. This elevates the flour from a mere staple to a functional food ingredient, contributing to wellness beyond basic nutrition. Gongmi flour possesses a respectable antioxidant profile, but the addition of ginger, with its high concentration of phenolic compounds, creates a synergistic effect. The functional properties are physically altered. The ginger powder, with its different fiber structure and chemical composition, likely influences the hydration properties of the blend.

### 2.3. Impact of Qingke Noodles Volatile Profiles and Quality

Qingke (highland barley) noodles represent a traditional Tibetan food product with unique characteristics influenced by the volatile profile of the base grain. Research on qingke noodles, a traditional Tibetan food made from highland barley, provides valuable insights into the volatile compounds present in highland barley and how they might influence noodle products [42]. The study identified 346 volatile compounds and 60 aroma active compounds in novel qingke noodles as shown in Table 3, with 14 compounds having particularly high flavor dilution factors and odor activity values. Although traditional and novel qingke noodles shared 39 volatile compounds, the novel version exhibited more than three times the overall concentration of volatile compounds, resulting in a richer flavor profile with more intense fruity, floral, and acidic aromas.

#### 2.3.1. Analysis of Volatile and Functional Compounds in Qingke Flours

The volatile profile of qingke flour is complex and consists of numerous compounds that collectively contribute to its distinct aroma characteristics. Through gas chromatography mass spectrometry (GC-MS) analysis, we have identified key volatile compounds that significantly impact the flour’s fragrance. The most prominent odor-active compounds include 7-hexadecenal (grassy), fumaric acid (cheesy), 2-pentylfuran (earthy), 1-pentanol (bready), pentanal (nutty), 1-octenol (mushroom-like), and E,E-2,4-decadienal (fried odor) as shown in Table 3 and Supplementary Figure S2. These compounds are primarily derived from lipid oxidation and amino acid metabolism pathways, with their concentrations varying significantly depending on processing methods and storage conditions. Qingke flour contains an impressive array of bioactive compounds that contribute to its health promoting properties. The most significant functional components include dietary fiber (particularly β-glucan), phenolic compounds, and minerals. The distribution of these compounds within the barley grain is heterogeneous, with the bran fraction containing substantially higher concentrations of most bioactive compounds compared to the endosperm. Research has shown that the contents of total protein, albumin, globulin, glutenin, free polyphenol, bound polyphenol, free flavone, bound flavone, and condensed tannin initially increase and then decrease with increasing milling rate. The formation of these volatile compounds is closely associated with the enzymatic activities of lipase and lipoxygenase, which catalyze the oxidation of unsaturated fatty acids, leading to the production of aldehydes, alcohols, and ketones that contribute to both desirable and undesirable aromas [43].

#### 2.3.2. The Volatile and Functional Profile of (90% Qingke + 10% Kudzu Root Flour)

While studying the volatile compound profile and elucidating the potential functional properties of a novel composite powder composed of 90% qingke (*Hordeum vulgare* L.) and 10% kudzu root (*Pueraria montana* var. *lobata*) flour, a total of 45 volatile compounds were identified, predominantly aldehydes, alcohols, ketones, furans, and pyrazines as shown in Table 3 and Supplementary Figure S3. The highland barley contributed key aroma compounds such as hexanal (grassy), 2-pentylfuran (earthy, beany), and nonanal (citrus, fatty), which are typical of cereal grains. The incorporation of kudzu root flour introduced distinct notes, potentially from specific alcohols and ketones associated with its unique herbal character. The functional profile, inferred from the identified compounds, suggests significant potential. Key findings include the presence of compounds with documented antioxidants (e.g., certain phenolic derivatives and furanones), antimicrobial (e.g., hexanal, nonanal), and potential health promoting properties. The synergistic combination of qingke dietary fiber and *β*-glucans with the isoflavonoid rich kudzu flour likely enhances the composite’s functional value, particularly for glycemic control, gut health, and antioxidant defense [44].

Aldehydes such as hexanal (grassy, green), heptanal (fatty, citrus), and nonanal (waxy, citrus) were among the most abundant. The presence of 2-pentylfuran, a well-known compound in barley, imparts the characteristic earthy, beany, and cereal-like aroma, which is a cornerstone of the product’s overall sensory identity [45]. Additionally, the detection of pyrazines (e.g., methylpyrazine) is significant, as these compounds are Maillard reaction products formed during any thermal processing (e.g., drying), contributing roasted, nutty notes that enhance the complexity of the aroma profile [46,47]. Contribution from kudzu root flour (10%): while the highland barley forms the aromatic backbone, the 10% kudzu root powder introduces subtle but distinctive nuances. The synergy between the two ingredients likely results in a masking or modification of any potential off-flavor, leading to a more palatable product. Beyond aroma, the identified compounds and the known composition of the raw materials point toward a promising functional food ingredient. Antioxidant potential: while GC-MS primarily identifies volatile compounds, the presence of certain furanones and phenolic aldehydes (e.g., vanillin) suggests inherent antioxidant capacity.

#### 2.3.3. The Volatile and Functional Profile of (90% Qingke Flour+10% Fishwort Root Flour)

**Figure 11:**
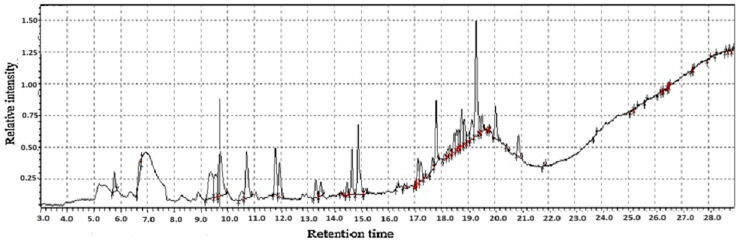
GC-MS two-way data in chromatography for (90% qingke flour + 10% fishwort root flour).

This study aimed to characterize the volatile compound profile and infer the functional potential of a novel composite flour blend comprising 90% qingke and 10% fishwort root (*Houttuynia cordata*) flour using gas chromatography mass spectrometry (GC-MS). The analysis revealed a complex volatile profile dominated by compounds derived from both constituents. Key compounds identified include 2-Undecanone and Decanoyl Acetaldehyde (major contributors from fishwort root, known for their antimicrobial and distinctive “fishy” aroma), and a suite of aldehydes (e.g., Hexanal, Nonanal), alcohols, and furans characteristic of cereal grains as shown in Table 3 and Figure 11, contributing green, fatty, and roasted notes. This unique profile could be desirable in specific savory baked goods, crackers, or noodles.

The most significant finding is the identification of key bioactive volatiles from fishwort root in the flour matrix, decanoyl acetaldehyde (also known as dodecanal) and 2-Undecanone are well documented in the scientific literature for their strong antimicrobial (against bacteria and fungi) and antioxidant activities [48]. These compounds, along with other detected terpenes and phenols, are associated with the traditional medicinal uses of fishwort root, including anti-inflammatory and potential antiviral effects.

#### 2.3.4. The Volatile and Functional Profile of (90% Qingke + 10% *Gastrodia elata* Blume Flour)

**Figure 12:**
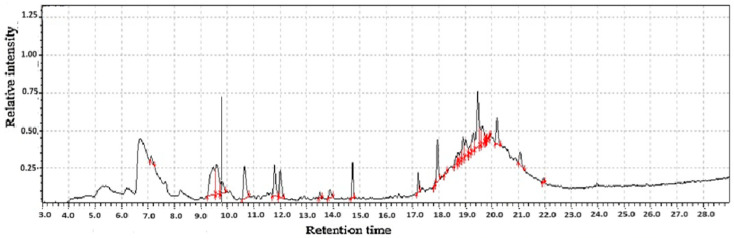
GC-MS two-way data in chromatography for (90% qingke flour + 10% *Gastrodia elata* blume flour).

The development of novel food products using composite flours is a growing trend, driven by the pursuit of enhanced nutritional value and unique sensory experiences. *Gastrodia elata* blume [1], a revered herb in traditional Chinese medicine (TCM), is prized for its bioactive compounds, particularly gastrodin, which is associated with neuroprotective and sedative effects [49,50]. Combining 90% qingke with 10% *GE* flour creates a promising functional ingredient. The GC-MS analysis provides critical insights into the flavor profile, potential health benefits, and overall quality of the composite flour. The volatile profile, crucial for the flour’s sensory characteristics, would be a blend of the nutty, roasted, and cereal like notes from qingke and the distinct earthy, herbal, and slightly sweet notes from *GE*. There are many compounds derived from qingke and *Gastrodia elata*; aldehydes (e.g., hexanal, heptanal, nonanal) these are lipid oxidation products and are among the most significant contributors to the green, grassy, and fatty notes characteristic of whole grains. Their presence indicates the freshness and lipid content of the barley. Pyrazines (e.g., 2,5-dimethylpyrazine, 2-ethyl-3,5-dimethylpyrazine) which are formed during Maillard reactions, impart roasted, nutty, and earthy aromas, which are highly desirable in cereal products. Alcohol (e.g., 1-hexanol, 1-octen-3-ol, citronellol, linalool, and benzyl alcohol) contributes green, earthy, sweet aromatic notes, and mushroom-like notes. Phenolic compounds (e.g., 4-hydroxybenzaldehyde, vanillin) the 4-hydroxybenzaldehyde is a direct precursor to gastrodin and has a characteristic aromatic scent. Vanillin contributes a familiar, sweet, vanilla-like aroma, which can pleasantly soften the cereal notes of the barley [51] as shown in Table 3 and Figure 12.

The 10% *GE* flour would not overpower the fundamental cereal character of the qingke but would significantly modify it. The nutty pyrazines from qingke would be complemented by the sweet, vanilla-like notes of vanillin and the aromatic phenolic character from *GE*, creating a more complex and sophisticated aroma profile compared to plain barley flour.

#### 2.3.5. The Volatile and Functional Profile of (90% Qingke + 10% *Polygonatum sibiricum* Redouté Flour)

This study characterized the volatile compound profile and inferred the functional potential of a novel composite flour consisting of 90% qingke and 10% *Polygonatum sibiricum* Redouté flour. Key volatiles identified included aldehydes such as hexanal and nonanal, contributing grassy, fatty, and waxy notes characteristic of whole grains; alcohols like 1-Octen-3-ol imparting earthy, and alkanes providing neutral background notes. Crucially, the presence of unique terpenoids and sterols, likely originating from the *Polygonatum sibiricum*, indicates a significant enrichment of the flour’s bioactive portfolio [52]. The results demonstrate that the incorporation of 10% *P. sibiricum* flour not only modifies the aromatic signature of qingke but also substantially enhances its functional profile, positioning this composite flour as a promising ingredient for developing innovative, health-oriented food products with a distinct sensory character.

The volatile organic compounds (VOCs) are responsible for the flour’s aroma and flavor. Aldehydes (e.g., hexanal, nonanal) are likely primary contributors from the qingke fraction, resulting from the oxidation of unsaturated fatty acids, they impart the characteristic green, grassy, and cereal notes [53,54]. Compounds in this class, such as phytosterols (e.g., β-sitosterol) or specific terpenes as shown in Table 3 and Supplementary Figure S4, are well documented bioactive constituents of *P. sibiricum*; they are not major contributors to aroma but are crucial for the flour’s functional value. The functional potential of this composite flour is inferred from the identified non-volatile and semi-volatile compounds that GC-MS can detect. The key functional enhancement comes from the 10% *P. sibiricum*. This herb is renowned in traditional medicine for its rich content of polysaccharides, saponins, flavonoids, and sterols [7,55,56]. The detection of sterols and potential saponin precursors via GC-MS confirms the successful integration of these bioactive ingredients into the flour noodles.

#### 2.3.6. The Volatile and Functional Profile of (90% Qingke + 10% Dried Ginger Flour)

The analysis revealed a complex volatile profile comprising a total of 45 identifiable compounds as shown in Table 3 and Supplementary Figure S5. The qingke base contributed predominantly to aldehydes such as hexanal and nonanal, associated with grassy, fatty notes, and alcohols like 1-hexanol, imparting fresh, plant-like aromas. The integration of 10% dried ginger flour significantly enriched the profile with potent bioactive ginger-derived compounds, most notably gingerols (e.g., 6-gingerol) and shogaols, which are responsible for ginger’s characteristic pungency and warmth. Additionally, key terpenes such as zingiberene, curcumene, and β-sesquiphellandrene the primary constituents of ginger’s essential oil were identified, contributing spicy, woody, and citrusy notes. The GC-MS analysis confirms that this composite flour is not merely a physical mixture but a functionally enhanced ingredient. This study demonstrates that the (90% qingke + 10% dried ginger) flour is a promising functional food ingredient with a distinct aroma profile and significant potential for health promoting applications.

## 3. Discussion

### 3.1. Utilization of Principal Component Analysis (PCA) to Discriminate Rice Noodles Based on Volatile Compound Profiles

Bioactive peptides from Gongmi proteins exhibit in vitro ACE-inhibitory activity [57] suggesting a potential mechanism for anti-hypertensive effects. Crucially, this bioactivity demonstrates stability across a range of thermal and pH conditions relevant to food processing [58,59], which analytically supports their feasibility as functional ingredients. Furthermore, Gongmi flours possess antioxidant capacities attributed to a composite of phenolic compounds, peptides, and polysaccharides. The new analysis focuses on the nature of the putative synergy: we propose that this enhanced antioxidant potential arises not merely from an additive effect, but from the distinct mechanisms (e.g., hydrogen donation, metal chelation) and differential bioavailability of each component class, which may provide sustained radical-scavenging activity across multiple physiological compartments [60]. This multi-mechanistic framework, supported by the composite data, positions Gongmi flours as a subject for further research into dietary strategies for mitigating oxidative stress-linked chronic diseases [61].

**Figure 13:**
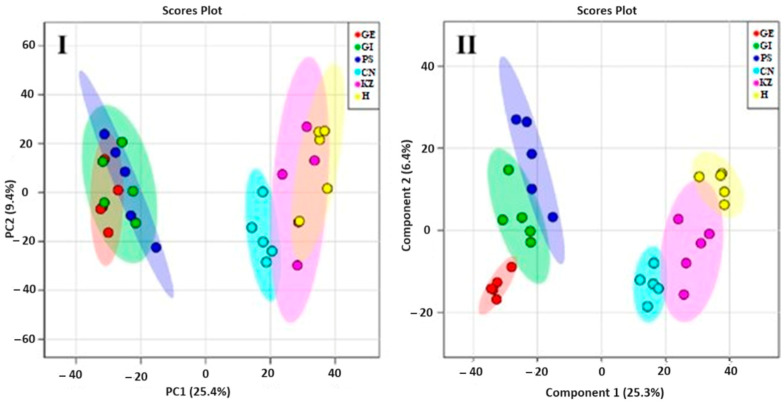
PCA score plots from the combined fraction’s mass spectra among six groups of volatile components.

While the total variance explained by the first two principal components (31.7%) may appear moderate as sown in Figure 13, we consider the model highly effective and informative for this specific analytical context. The variance distribution is typical for complex, multicomponent systems like food volatiles, where numerous low-concentration compounds contribute to the overall profile. Crucially, the score plot reveals strong, interpretable patterns: PC1 clearly separates the control from all fortified samples, confirming a significant baseline alteration, while PC2 successfully discriminates between fortificant types based on their distinct chemical signatures. The tight clustering of replicates validates the method’s robustness. Therefore, the PCA model effectively captures the most significant sources of variation driven by the fortification, providing clear and actionable insights into the volatile profile modifications [1]. The analysis moves beyond merely detecting changes; it categorizes the mode of aromatic modification. PC1 explains the magnitude of change from the control (the “if” of alteration), while PC2 explains the direction or quality of that change (the “how” or “type” of alteration). This two-tiered interpretation demonstrates that fortification first overrides the base aroma, and then imposes a new, ingredient-specific volatile profile classifiable by key chemical families (pungent/aromatic vs. earthy/green). This provides a predictive framework for estimating the sensory impact of new functional ingredients based on their known volatile chemistry. The PCA of volatile compounds reveals three primary scientific insights: PC1 (25.4% variance) primarily captures the fundamental difference between unfortified and fortified samples. The control group’s distinct separation, driven by higher levels of common cereal volatiles (e.g., hexanal), indicates that the addition of any functional ingredient significantly masks or alters the base rice noodle’s inherent aroma profile. PC2 (9.4% variance) reveals that fortificants impart distinct and characteristic signatures. The clear separation of ginger/*Gastrodia elata* (upper quadrants) from kudzu/Houttuynia (lower quadrants) along this axis demonstrates that the volatile fingerprints are not just different from the control but are also chemically distinct from each other. This suggests that PCA can discriminate between different classes or types of functional ingredients based on their volatile metabolites. The tight, reproducible clustering of samples within each fortified group confirms the analytical precision of the method and the consistent impact of each ingredient [1].

The PCA model successfully demonstrates that the volatile compound profile is a powerful metric for rapidly discriminating and authenticating rice noodles based on their botanical fortification. The distinct positioning of each group confirms that Gongmi flour (CN), kudzu (KZ), Houttuynia (H), *Gastrodia elata* blume [1], *Polygonatum sibiricum* (PS), and ginger (GI) each contribute a characteristic and identifiable aroma profile.

**Figure 14:**
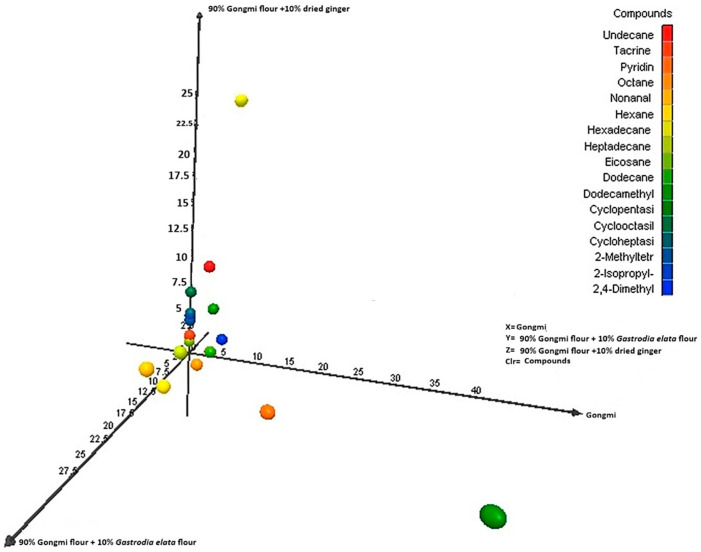
The diversity of rice noodles according to various volatile components. The principal components analysis (PCA) plots represent the distribution of different volatiles.

The core message of the PCA is to show how the addition of dried ginger and *Gastrodia elata* alters the volatile fingerprint of the base Gongmi rice noodle as shown in Figure 14.

Group X (100% Gongmi) will likely cluster around the center or a specific region, representing the baseline volatile profile. This profile is typically characterized by neutral, grain-like, and slightly sweet aromas from compounds like hexanal (grassy), 2-pentylfuran (beany), and 1-octen-3-ol (mushroom-like). While, group Z (ginger 10%) will form a distinct cluster, significantly separated from group X along one of the PCs (like PC1), this separation is driven by the potent, characteristic volatiles of ginger, such as terpenes, aldehydes, and alcohols. The ginger’s volatiles are strong and distinct, overpowering or significantly modifying the base rice noodle profile. Group X (*Gastrodia elata* 10%) will form another distinct cluster, separated from both Y and X, possibly along PC2. *Gastrodia elata* has a unique, medicinal, and earthy aroma profile [62,63], key volatiles might include phenolic compounds and benzenoids [64]. Unique esters and alcohols contribute to its herbal, woody, and slightly sweet notes. This creates a volatile profile distinctly different from both the base noodle and the *Gastrodia elata* infused one.

**Figure 15:**
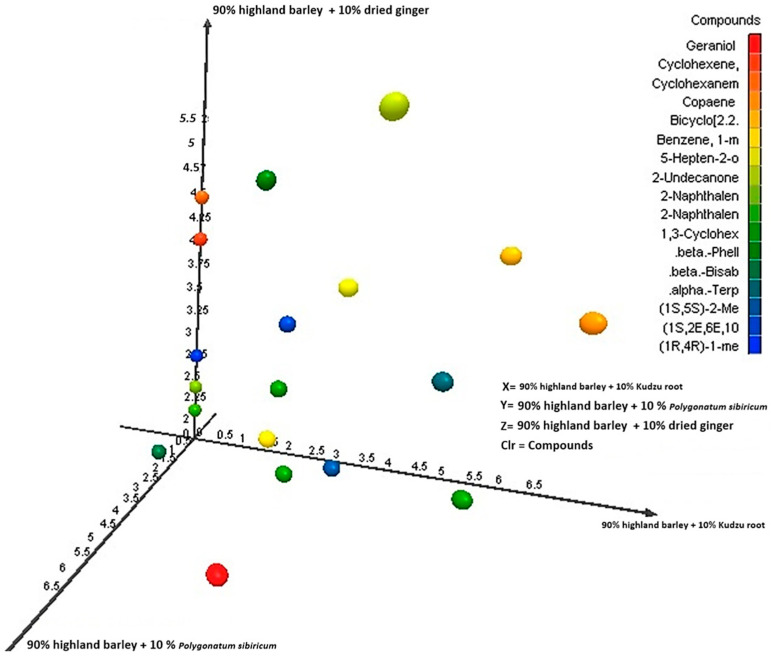
The diversity of qingke noodles according to various volatile components. The principal components analysis (PCA) plots represent the distribution of different volatiles.

**Figure 16:**
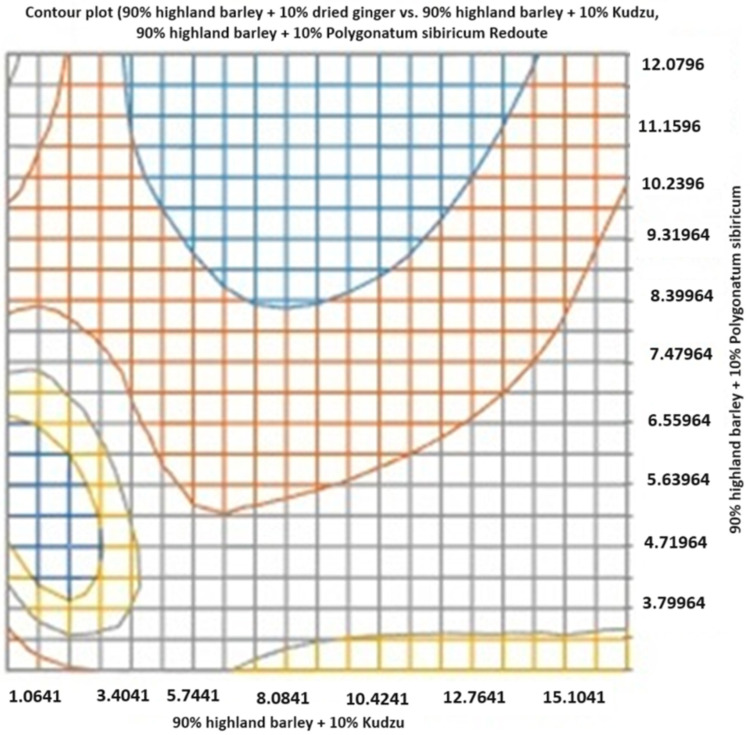
The distribution of the relative volatile components for the proposed qingke noodles.

Relationship between axes (X, Y, Z compositions) and volatiles: The distance between clusters directly relates to volatile dissimilarity. The greater distance (e.g., between X and Y), the more different their overall aroma, if groups X and Z are on opposite sides of group Y, it indicates that ginger and *Gastrodia elata* impart fundamentally different types of volatile compounds to the noodles. Inferences from the PCA plot—successful flavor infusion: the clear separation confirms that the volatile compounds from both ginger and *Gastrodia elata* are present and detectable in the cooked noodles. Aroma diversity: the plot visually demonstrates the diversity mentioned in the title. The three noodle types occupy different sensory spaces providing potential for distinct products; the results suggest that these additions can create noodles with unique and identifiable aroma signatures, appealing to different consumer preferences (spicy/warm vs. herbal/earthy). Impact of base flour: the fact that all samples share a base (Gongmi flour) is why they may not be scattered wildly; they are variations on a common theme. The base flour volatiles contribute to the shared background. The primary goal of this PCA plot is to compare the overall volatile compound composition (the aroma fingerprint) of the three noodle formulations as shown in Figure 15 and Figure 16. It answers the question: How do the different added flours (kudzu root, *Polygonatum sibiricum*, and dried ginger) alter the aromatic profile of the base highland barley (qingke) noodles?

The description indicates the axes (X, Y, Z) are not standard PCA axes (PC1, PC2) but are labels for the three specific noodle samples. This is a crucial point. In a standard PCA biplot, the points represent individual noodle samples (likely with replicates for each type). The axes (PC1 and PC2) are synthetic, statistical axes that explain the maximum variance in the volatile compound data. PC1 (horizontal) explains the largest direction of difference, and PC2 (vertical) explains the next largest. The X, Y, Z labels would therefore be group labels for the clusters of points corresponding to each noodle type, plotted along the PC1/PC2 axes. Therefore, the relationship is: X-axis (PC1) and Y-axis (PC2) represent the dominant patterns of variation in volatile compound concentrations across all samples. Groups X, Y, Z: the three noodle formulations, whose positioning on the PC1/PC2 plane reveals their aromatic relationships. Contribution of volatiles (loading vectors), the plot often includes vectors (lines) or labels for the most influential volatile compounds (e.g., aldehydes, alcohols, ketones, terpenes). Interpretation, a noodle cluster located in the direction of a specific vector, is rich in that volatile compound. For example, if the Z (ginger 10%) cluster is positioned in the direction of vectors labeled zingiberene/citral it confirms these ginger-specific terpenes are key drivers of its unique profile. If X (kudzu 10%) and Y (*Polygonatum* 10%) share a direction for a vector like hexanal (a common lipid oxidation compound), it might suggest their aromas share some common grassy/earthy notes from the base flour.

### 3.2. Future Directions and Applications

The discussion highlights a strategic research framework for optimizing flavor and functionality in noodles incorporating rooted plant flours. The primary analytical insight is that moving beyond mere volatile profiling to a dynamic, process-oriented understanding is crucial. This involves systematically mapping volatile compound evolution under specific noodle-processing conditions to enable predictive flavor design. A key advancement would be investigating not just individual compounds, but their synergistic or antagonistic interactions within composite flour matrices to engineer a balanced sensory profile. Furthermore, a significant analytical pivot is proposed: to treat volatile compounds not only as flavor agents but as bioactive constituents. This requires studying their transformation during processing and digestion, and quantitatively linking specific volatile profiles to demonstrated in vitro or in vivo functional benefits, such as antioxidant capacity or digestive modulation. This integrated approach would transform volatile analysis from a descriptive tool into a targeted strategy for developing noodles with superior, evidence-based flavor and health attributes [65,66].

From an application perspective, the development of processing technologies that maximize the retention of desirable volatile compounds while ensuring satisfactory texture and cooking quality is essential [67,68]. The successful incorporation of kudzu root dietary fiber at 10% level in biscuits while maintaining attractive color, crisp texture, and unique flavor suggests similar potential for noodle products [3,69,70,71]. Additionally, the economic advantages of using *Gastrodia elata* blume flour and tuber flours highlight the commercial viability of these ingredients for developing value-added functional noodle products that can benefit both producers and consumers.

This research establishes a novel framework for functional food development by innovatively incorporating rooted plant flours into noodle matrices, which concurrently improves nutritional density and sensory appeal. A key analytical advancement is the use of in silico molecular docking to predict the binding affinities and interactions of key volatile organic compounds (VOCs) with physiological targets such as the angiotensin-converting enzyme (ACE). This provides a mechanistic, theoretical basis for the proposed health benefits, moving beyond mere correlation to hypothesize specific bioactive pathways. The study thus offers a strategic, evidence-based model for designing next-generation staple foods that effectively reconcile palatability with targeted physiological efficacy, with implications for personalized nutrition and sustainable food systems.

## 4. Conclusions

The GC-MS analysis of volatile compounds clearly demonstrates that the strategic incorporation of functional root flours specifically Gastrodia tuber and *Polygonatum sibiricum* significantly and uniquely modulates the aroma profiles of both rice (Gongmi) and highland barley (qingke) noodles. Among the 12 groups were used and analyzed, the formulations of Gongmi flour 90% + Gastrodia tuber flour 10% and Gongmi flour 90% + *Polygonatum sibiricum* flour 10% are of particular importance. These blends successfully enrich the relatively neutral aroma of rice noodles with distinct, valuable volatile notes from the medicinal roots such as earthy, sweet, or herbal compounds without overwhelming the base character. This indicates a successful creation of a palatable, value-added functional food that leverages the traditional medicinal cachet of these roots in a staple format. Similarly, the formulations qingke flour 90% + Gastrodia tuber flour 10% and qingke flour 90% + *Polygonatum sibiricum* flour 10% showcase a crucial synergistic interaction. The inherently nutty, roasted aroma of qingke noodles interacts with the volatile compounds from the root flours, potentially creating more complex and balanced aromatic profiles than simple addition would predict. This synergy is key to developing appealing functional foods from whole grain qingke, enhancing its health benefits with those of the medicinal roots while maintaining or even improving its sensory appeal. The overall importance of these four specific formulations lies in their proven potential to bridge the gap between traditional functional ingredients and modern staple foods. The GC-MS data provides a scientific basis for aroma design, confirming that a 10% incorporation level is effective in introducing characteristic volatile markers of Gastrodia tuber and *Polygonatum sibiricum*. This paves the way for developing next-generation nutritious noodles that offer not only enhanced phytochemical content but also distinctive and acceptable aromatic signatures, meeting the growing consumer demand for health-promoting foods with appealing sensory qualities. Future research should focus on mapping the volatile compound profiles of various rooted plant flours, understanding their interactions in composite flour systems, and elucidating the health implications of these compounds and their transformation products.

## 5. Materials and Methods

### 5.1. Plant Materials

This investigation was carried out at the Yunnan Academy of Agricultural Science (YAAS), during 2025 seasons, at Kunming city, Yunnan province, China. Gongmi flour is a processed product made from Gongmi 3, a type of rice produced in Lianghe County, Dehong Prefecture, Yunnan Province; the process involves soaking the rice in water for 12 h, then grinding it into fine powder. qingke powder is made from highland barley Yunke 5, which is planted in the research and production base of Songming, Yunnan Academy of Agricultural Sciences, it is processed by soaking for 12 h and grinding. Five functional food flours, kudzu root (*Puerariae lobatae* Radix), fishwort root (*Houttuynia cordata* Thunb.), gastrodia tuber (*Gastrodia elata* blume), *Polygonatum sibiricum* (*Polygonatum sibiricum* Redouté), and dried ginger (*Zingiberis rhizoma*) were purchased from Jianzhijia Pharmacy in Kunming, China. The recipes for rice noodles and Qingke noodles are as follow:
**Rice Noodles****Qingke Noodles**Gongmi flour 100%Qingke flour 100%Gongmi flour 90% + kudzu root flour 10%Qingke flour 90% + kudzu root flour 10%Gongmi flour 90% + fishwort root flour 10%Qingke flour 90% + fishwort root flour 10%Gongmi flour 90% + gastrodia tuber flour 10%Qingke flour 90% + gastrodia tuber flour 10%Gongmi flour 90% + *Polygonatum sibiricum* flour 10%Qingke flour 90% + *Polygonatum sibiricum* flour 10%Gongmi flour 90% + dried ginger flour 10%Qingke flour 90% + dried ginger flour 10%

The recipes of 12 kinds of functional rice noodles and highland barley noodles are listed in the above table. The rice noodles or qingke noodles made from pure Gongmi flour or qingke flour were used as control. Five kinds of functional rice noodles were developed with 90% Gongmi flour and another five kinds of functional food using rice noodle machines. Similarly, five kinds of functional qingke noodles were developed with 90% qingke flour and another five kinds of functional food using rice noodle machines.

### 5.2. Methodology

Advanced analytical techniques have revolutionized our ability to identify and characterize volatile flavored compounds in complex food matrices. Gas chromatography spectrometry (GC-MS) has emerged as a particularly valuable tool for analyzing volatile organic compounds in food products, offering high sensitivity and the ability to detect compounds at very low concentrations without extensive sample preparation [72]. This technique has been successfully applied to study volatile profiles in various flour-based products, including rice and qingke flour and noodles. All raw materials and their prepared fresh rice noodles or fresh qingke noodles were repeated three times for technical replicates, and each replicate was accurately weighed at 10 g and placed into a 250 mL conical flask, sealed, and heated at 50 °C for 5 min to extract volatile flavor components using the JE2002 electronic balance (Shanghai Shangpu Instrument Equipment Co., Ltd., Shanghai, China). During the heating process, it was shaken from time to time to ensure that the sample in the bottle was evenly heated, the sample was placed in a sealed flask for heating, and the SPME extraction head was inserted through the holes in the sealing film to complete the sample extraction process via static headspace extraction. The solid-phase microextraction instrument includes a Stableflex solid-phase microextraction fiber head (white) and a headspace extraction bottle (Shanghai Anpu Experimental, Shanghai, China). The injector program AOC-20i+s solvent rinse cycles in pre-injection: (R): Three times; solvent rinse cycles in post-injection. (P): Three times; sample rinse cycles. (S): Three times; plunger flow rate. (L): high; viscosity compensation time. (V): 0.2 s; plunger injection speed. (I): High; injector injection speed. (Y): High; injection method of normal. (M): 0. SPL1. Temperature: 280 °C; injection method: split; injection time: 2.00 min; carrier gas: He; pressure: 57.4 kPa; total flow rate: 14 mL/min; chromatographic column flow rate: 1.0 mL/min; line speed: 36.5 cm/s; purge flow rate: 3.0 mL/min; split ratio: 10.0; high-pressure injection: off; save carrier gas: off. Chromatography column temperature: 60.0 °C; equilibrium time (it takes three minutes for the chromatography column to rise to 60°): 3 min; chromatographic column information: Rtx-5MS; Column ID: DB-5MS (30 m × 0.25 mm × 0.25 µm); installation date: 5 June 2025; length: 30.0 m; inner diameter: 0.25 mm ID; membrane thickness: 0.25 μm. Ion source temperature (O): 230 °C; interface temperature (T): 280 °C; solvent delay time (S): 3 min; detector voltage: relative to tuning results; no CID gas used for analysis (Q3 Scan); threshold (H): 0.2 kV; GC program duration: 45.33 min Dichloromethane, ethyl acetate, or hexane for non-polar compounds (terpenes, phenols). Ethyl acetates for polar compounds (esters, alcohols, acids). Extraction Steps: Mix sample with solvent in a separatory funnel, shake vigorously, and allow phase separation. Collect the organic layer, dry (anhydrous Na_2_SO_4_), and concentrate under nitrogen before GC-MS analysis. Instrumental conditions: column, DB-5MS (30 m × 0.25 mm × 0.25 µm) for broad-range separation. Oven Program, 40 °C (hold 2 min) → 10 °C/min → 250 °C (hold 5 min). Ionization, EI mode (70 eV). Detection: Full scan (m/z 35–350) or SIM for targeted compounds. Optimizing extraction parameters (time, temperature, pH) is critical for accurate GC-MS analysis in alcoholic beverage quality control and flavor research. Instrument program setting: chromatographic column temperature: 60 °C; SPLI temperature: 280; equilibrium time: 3min; column temperature chamber temperature program: 60 °C, keep for one minute; rate 7 °C/min, 180 °C keep for 0 min, speed 3 °C/min, 210 °C keep for 1 min, speed 15 °C/min, 280 °C keep for 5 min. To translate spectra into an ASCII format, AMIX program (version 3.9.7, BrukerBioSpin, Kunming, China) is widely used [73], the peak is integrated into a small bin (bucket) of 0.01 mg kg^−1^ in this process. The obtained data was subjected to ANOVA using SAS software (Version 9.1; SAS Institute, Cary, NC, USA). The principal component analysis (PCA) was done by using Prcomp and HCPC functions and FactoMineR and FactoExtra packages in RStudio (Version 2026.01.2+418). The AMIX-identified bucket was important.

## Figures and Tables

**Table 1:** Mean performance of concentration for the best compounds which are selected among more than 150 compounds in different rooted plant flours for functional foods.

Compounds	Dried Ginger Flour		*Polygonatum sibiricum* Redoute Flour		
	Concentration	Retention Time	Compounds	Concentration	Retention Time
5-Heptenone	3.903 ± 0.43	9.83	Furan	3.139 ± 0.04	8.58
β-Phellandrene	4.712 ± 1.23	11.65	Nonanal	3.857 ± 0.68	10.24
Bicycloheptanol	4.146 ± 0.67	16.57	Chrysanthenol	2.093 ± 1.09	10.83
α-Terpineol	3.276 ± 0.20	17.26	Octadecane	3.375 ± 0.20	14.19
Geraniol	2.201 ± 1.28	18.81	Cyclodecadiene	6.285 ± 3.11	14.29
Undecanone	5.125 ± 1.65	20.05	Bicyclononane	6.141 ± 2.96	14.79
Copaene	3.853 ± 0.38	22.68	Cyclohexene	4.003 ± 0.82	14.90
Cyclohexadiene	2.950 ± 0.53	23.23	Cyclopropazulene	2.680 ± 0.50	15.04
Tetramethylbicyclo	3.468 ± 0.01	24.18	Benzene	2.177 ± 1.00	15.40
Benzene	2.543 ± 0.93	25.54	Naphthalene	2.995 ± 0.18	15.62
β-Bisabolene	2.108 ± 1.37	26.79	Isospathulenol	2.237 ± 0.94	16.95
Cyclohexanemethanol	4.489 ± 1.01	27.46	Alloaromadendrene	2.334 ± 0.84	18.17
Naphthalenemethanol	2.428 ± 1.05	29.79			
Compounds	*Gastrodia elata* blume		Compounds	Kudzu root flour	
	Concentration	Retention time		Concentration	Retention time
Benzaldehyde	3.305 ± 0.13	8.27	Furfural	34.126 ± 27.3	13.56
Furan	5.513 ± 2.34	8.58	Ethanone	4.557 ± 2.27	16.78
Cresol	4.734 ± 1.56	9.99	Furancarboxaldehyde	6.250 ± 0.58	18.90
Heptadecane	2.246 ± 0.93	10.14	Benzeneacetaldehyde	4.069 ± 2.76	22.25
Dodecane	3.883 ± 0.71	11.56	Nonanal	5.510 ± 1.32	24.54
Benzene	6.323 ± 3.15	12.33	Hydroxymethylfurfural	4.330 ± 2.50	29.24
Cyclohexasiloxane	2.261 ± 0.91	12.72	Dimethylphthalate	2.945 ± 3.88	35.81
Hexadecane	2.181 ± 0.99	12.90	Compounds	Fishwort root flour	
Octenal	5.186 ± 2.01	13.90		Concentration	Retention time
Alloaromadendrene	2.461 ± 0.71	15.62	Bicycloheptane	4.349 ± 0.38	8.55
2,4-Ditertbutylphenol	3.187 ± 0.01	15.62	Undecanone	23.298 ± 18.57	12.91
			Dodecanone	4.252 ± 0.48	13.97
			Tridecanone	2.554 ± 2.17	15.28

Note. Data averaged over three replicates, values = mean ± standard deviation.

**Table 2:** Mean performance of concentration for the best compounds which are selected among more than 120 compounds of rice noodle flours for functional foods.

Compounds	Gongmi 90% + Kudzu 10%		Compounds	Gongmi 90% + *Poly. sib.* 10%		Compounds	Gongmi 90% + Dried Ginger 10%	
	Conc.	Ret. Time		Conc.	Ret. Time		Conc.	Ret. Time
Pyridin	17.72 ± 12.6	9.49	Formic acid	1.33739 ± 4.28	7.677	Hexanal	1.58938 ± 3.56	6.678
Hexane	4.52 ± 0.59	12.17	Cyclotetrasiloxane	12.44187 ± 6.83	9.461	Benzaldehyde	2.11056 ± 3.12	9.483
Undecane	2.48 ± 2.63	12.35	Octanal	5.0352 ± 0.58	10.009	5-Hepten	9.46302 ± 4.23	9.669
Nonanal	12.48 ± 7.37	13.89	1-Octanol	1.51036 ± 4.11	11.191	2-Cyclohexenol	5.25086 ± 0.02	10.733
Cyclopentasiloxane	7.83 ± 2.72	14.66	Nonanal	32.58256 + 26.97	11.797	Nonanal	3.66869 ± 1.57	11.795
Heptadecane	1.24 ± 3.87	17.12	Cyclopentasiloxane	3.97087 ± 1.64	12.013	Bicyclol	4.26028 ± 0.97	13.284
Octane	1.38 ± 3.73	17.50	1-Nonanol	1.42758 ± 4.19	12.92	Octadecane	1.0795 ± 4.16	16.515
Dodecane	1.59 ± 3.52	18.76	Decanal	1.47544 ± 4.14	13.513	1,5-Cyclodecadiene	1.64872 ± 3.59	17.236
2,4-Dimethyldodecane	2.45 ± 2.66	19.05	Cyclohexasiloxane	3.40316 ± 2.21	14.737	Trimethyltridecane	1.93249 ± 3.30	17.385
Hexadecane	4.59 ± 0.52	19.44	Cycloheptasiloxane	2.75474 ± 2.86	17.218	Benzene	24.63025 ± 19.40	17.938
Dodecamethyl	7.83 ± 2.72	20.04	Eicosane	1.61016 ± 4.01	17.372	1,3-Cyclohexadiene	4.9379 ± 0.30	18.117
2-Isopropyl-methyl	1.11 ± 4.00	20.22	Heptadecane	3.73587 ± 1.88	17.96	Caryophyllene	4.01387 ± 1.22	18.33
2-Methyltetracosane	1.21 ± 3.90	23.30	Squalane	1.71477 ± 3.90	18.921	Naphthalene	3.47143 ± 1.76	18.503
Cycloheptasiloxane	4.73 ± 0.38	24.58	11-Methyltricosane	1.23741 ± 4.38	19.04	Cyclohexene	4.22076 ± 1.01	18.62
Eicosane	2.73 ± 2.28	25.29	Hexadecane	7.42585 ± 1.81	19.477	Epoxylanostanol	1.27396 ± 3.96	19.33
Cyclooctasiloxane	1.60 ± 3.51	28.45	Cyclooctasiloxane	3.02556 ± 2.59	19.641	Hexadecane	6.42051 ± 1.19	19.49
Tacrine	1.83 ± 3.28	32.54	Eicosane	3.0589 ± 2.56	20.211	Eicosane	1.88491 ± 3.35	21.064
Compounds	Gongmi 90% + Fishwort Root 10%		Compounds	Gongmi 100%		Compounds	Gongmi 90% + *Gast. elata* 10%	
	Conc.	Ret. Time		Conc.	Ret. Time		Conc.	Ret. Time
Silanediol	2.99 ± 1.63	4.98	Hexanal	16.53 ± 9.40	12.11	Silanediol	14.92 ± 7.23	4.85
Hexanal	6.33 ± 1.71	6.09	1-Octenol	3.42 ± 3.71	19.68	Acetoin	14.90 ± 7.21	5.04
Cyclotrisiloxane	6.89 ± 2.27	6.28	Furan	3.46 ± 3.67	20.01	2,3-Butanediol	1.52 ± 6.17	5.82
Heptanal	1.16 ± 3.46	7.71	1-Octanol	3.82 ± 3.31	23.21	Hexanal	19.75 ± 12.06	5.95
Benzaldehyde	12.67 ± 8.05	9.41	Nonanal	36.84 ± 29.71	24.53	Benzaldehyde	27.17 ± 19.48	9.33
Bicyclool	1.88 ± 2.74	10.00	2-Nonenal	4.28 ± 2.85	26.69	Nonanal	12.30 ± 4.61	13.83
Octanal	4.83 ± 0.21	10.42	5-Amino-3TMS	3.86 ± 3.27	31.10	Cyclohexasiloxane	5.93 ± 1.76	20.03
Nonanal	12.69 ± 8.07	13.88	Hexadecane	4.86 ± 2.27	31.35	Cycloheptasiloxane	3.50 ± 4.19	24.56
Alloaromadendrene	3.86 ± 0.76	14.65	Butyl benzoate	5.28 ± 1.85	33.77			
Borneol acetate	2.11 ± 2.51	19.88	Heptadecane	6.63 ± 0.50	34.26			
2-Undecanone	2.33 ± 2.29	19.97	Cycloheptasiloxane	3.72 ± 3.41	35.63			
Hexadecane	1.19 ± 3.43	23.87						
Benzene	1.18 ± 2.4	25.26						
Heptadecane	2.22 ± 4.6	25.57						

Note. Data averaged over three replicates, values = mean ± standard deviation. *Poly. sib.*: *Polygonatum sibiricum* Redouté; *Gast. elata*: *Gastrodia elata* blume.

**Table 3:** Mean performance of concentration for the best compounds which are selected among more than 400 compounds of qingke noodle flours for functional foods.

Compounds	Qingke 90% + Kudzu 10%		Compounds	Qingke 90% + *Poly. sib.* 10%		Compounds	Qingke 90% + Dried Ginger 10%	
	Conc.	Ret. Time		Conc.	Ret. Time		Conc.	Ret. Time
Hexanal	16.535 ± 13.16	6.69	Cyclotetrasiloxane	9.317 ± 3.23	9.33	Cyclotrisiloxane	1.439 ± 4.92	6.95
Heptanal	1.092 ± 2.29	8.24	1,3-Hexadiene	7.781 ± 1.69	10.73	Hepten	3.332 ± 3.02	9.69
Cyclotetrasiloxane	4.079 ± 0.70	9.31	Nonanal	5.951 ± 0.41	11.81	Nonanal	5.226 ± 1.13	10.69
2-Pentylfuran	2.363 ± 1.02	9.73	Cyclopentasiloxane	11.955 ± 5.87	11.94	1-Hexanol	1.417 ± 4.94	11.73
1,3-Hexadiene	7.226 ± 3.85	10.68	Decanal	4.625 ± 1.46	13.51	6-Gingerol	3.217 ± 3.14	13.26
Nonanal	2.599 ± 0.78	11.77	Cyclohexasiloxane	7.929 ± 1.84	14.66	α-Terpineol	1.099 ± 5.26	13.53
Methylpyrazine	3.723 ± 0.34	11.93	Cycloheptasiloxane	2.879 ± 3.21	17.13	1,5-Cyclodecadiene	1.132 ± 5.22	17.10
Cyclohexasiloxane	2.367 ± 1.01	14.64	Eicosane	4.643 ± 1.45	19.31	Benzene	29.094 ± 22.74	17.80
Hexadecane	1.632 ± 1.75	19.29	Cycloheptatrien	7.156 ± 1.07	22.19	1,3-Cyclohexadiene	12.696 ± 6.34	17.97
Cycloheptatrien	1.254 ± 2.13	22.19	Docosa-2,21-dione	3.961 ± 2.13	23.88	Zingiberene	8.761 ± 2.41	18.17
Heptasiloxane	1.064 ± 2.32	24.09	Heptasiloxane	10.872 ± 4.78	24.11	Hexenal	5.946 ± 0.41	18.35
			Cyclooctasiloxane	2.091 ± 4.00	28.13	β-Sesquiphellandrene	9.244 ± 2.89	18.45
Compounds	Qingke 90% + Fishwort Root 10%		Compounds	Qingke flour 100%		Compounds	Qingke 90% + *Gast. elata* 10%	
	Conc.	Ret. Time		Conc.	Ret. Time		Conc.	Ret. Time
Silanediol	1.887 ± 2.49	5.76	1-pentanol	2.701 ± 0.19	9.96	Pyridin	13.132 ± 7.73	9.46
Hexanal	4.106 ± 0.27	6.73	1,3-Hexadiene	3.245 ± 0.73	10.80	Octenol	11.502 ± 6.10	9.59
4-tert-Octylphenol	2.475 ± 1.90	6.82	E,E-2,4-decadienal	3.045 ± 0.53	11.89	Furan	2.756 ± 2.64	9.78
Cyclotetrasiloxane	5.138 ± 0.76	9.36	Cyclopentasiloxane	2.501 ± 0.01	11.95	1,3-Hexadiene	8.894 ± 3.49	10.66
Benzaldehyde	2.989 ± 1.39	9.55	pentanal	2.670 ± 0.15	12.12	Nonanal	7.447 ± 2.05	11.79
Butanoic acid	9.919 ± 5.54	9.71	Trifluoroacetoxytetradecane	2.272 ± 0.24	12.21	Cyclopentasiloxane	5.828 ± 0.43	12.01
1,3-Hexadiene	6.463 ± 2.09	10.71	Sulfurous acid	1.689 ± 0.83	14.47	Decanal	1.108 ± 4.29	13.51
Nonanal	5.259 ± 0.88	11.79	Cyclohexasiloxane	1.663 ± 0.85	14.68	Dianhydroglucopyranose	2.799 ± 2.60	13.88
Cyclopentasiloxane	4.051 ± 0.32	11.95	2-pentylfuran	1.507 ± 1.01	17.15	Cyclohexasiloxane	4.650 ± 0.75	14.73
Borneol	2.172 ± 2.20	13.30	Heptasiloxane	2.289 ± 0.23	23.91	Cycloheptasiloxane	2.903 ± 2.50	17.22
Decanoyl Acetaldehyde	1.699 ± 2.68	13.49	Fumaric acid	2.167 ± 0.35	23.99	Heptadecane	6.741 ± 1.34	17.95
Cyclohexasiloxane	3.386 ± 0.99	14.66	7-Hexadecenal	1.540 ± 0.98	24.79	Decanol	1.245 ± 4.15	18.63
2-Undecanone	7.342 ± 2.97	14.89	Diisooctyl phthalate	5.416 ± 2.90	24.92	Dodecane	1.188 ± 4.21	18.72
Cycloheptasiloxane	1.879 ± 2.50	17.13	Di-Cyclohexylieden	2.279 ± 0.24	27.65	Pentadecane	3.069 ± 2.33	18.91
Eicosane	2.065 ± 2.31	17.23	1-octenol	1.987 ± 0.53	27.84	11-Methyltricosane	2.098 ± 3.30	19.02
Heptadecane	4.579 ± 0.20	17.81	Pyridine	2.110 ± 0.41	27.84	Hexadecane	8.784 ± 3.38	19.47
11-Methyltricosane	1.593 ± 2.78	18.89	Deoxyartemisinin	1.549 ± 0.97	28.26	Cyclooctasiloxane	3.410 ± 1.99	19.64
Hexadecane	9.513 ± 5.14	19.31				Trimethyltridecane	1.843 ± 3.56	21.07

Note. Data averaged over three replicates, values = mean ± standard deviation. *Poly. sib.*: *Polygonatum sibiricum* Redouté; *Gast. elata*: * Gastrodia elata* blume.

## Data Availability

The original contributions presented in this study are included in the article/Supplementary Materials. Further inquiries can be directed to the corresponding authors.

## Supplementary Materials

The following supporting information can be downloaded at: https://www.mdpi.com/article/10.3390/molecules31081348/s1, Figure S1: GC-MS two-way data in chromatography for (90% Gongmi flour + 10% *Gastrodia elata* Blume flour); Figure S2: GC-MS two-way data in chromatography for qingke flour; Figure S3: GC-MS two-way data in chromatography for (90% qingke flour + 10% Kudzu rootflour); Figure S4: GC-MS two-way data in chromatography for (90% highland barley flour + 10% *Polygonatum sibiricum* Redouté flour); Figure S5: GC-MS two-way data in chromatography for (90% qingke flour + 10% dried ginger flour).

## Abbreviations

Principal Component Analysis (PCA), Gongmi flour (CN), Kudzu (KZ), Houttuynia (H), *Gastrodia elata* blume [1], *Polygonatum sibiricum* (PS), and Ginger (GI).
