# Supplementary material for: GC-MS Analysis of Volatile Differences in Rice and Qingke Noodles Formulated with Functional Root Plant Flours
Source: Molecules. 2026 Apr 20;31(8):1348. doi: 10.3390/molecules31081348 (PMC13119050; doi:10.3390/molecules31081348)
Supplement: Supplementary file 1 [file molecules-31-01348-s001.zip › molecules-4115824-supplementary.pdf]

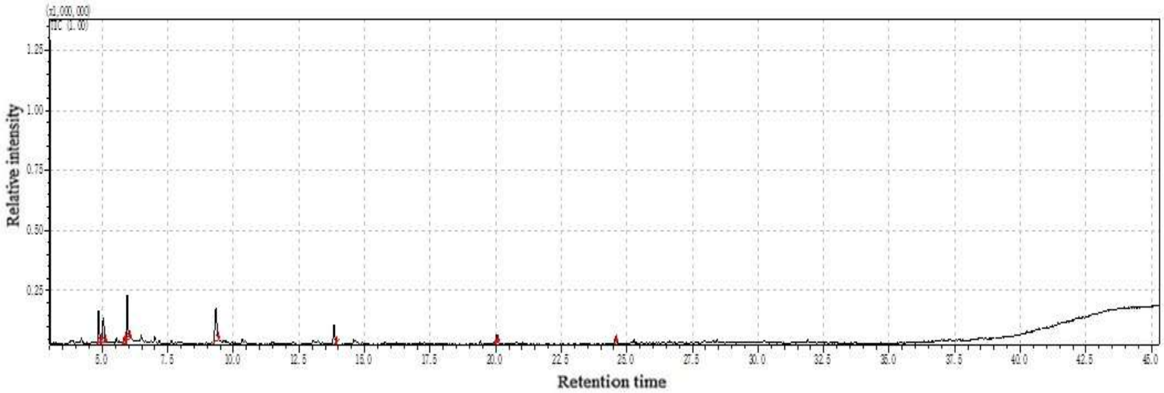

Supplementary Figure S1. GC-MS two-way data in chromatography for (90% Gongmi flour+10% *Gastrodia elata* Blume flour)

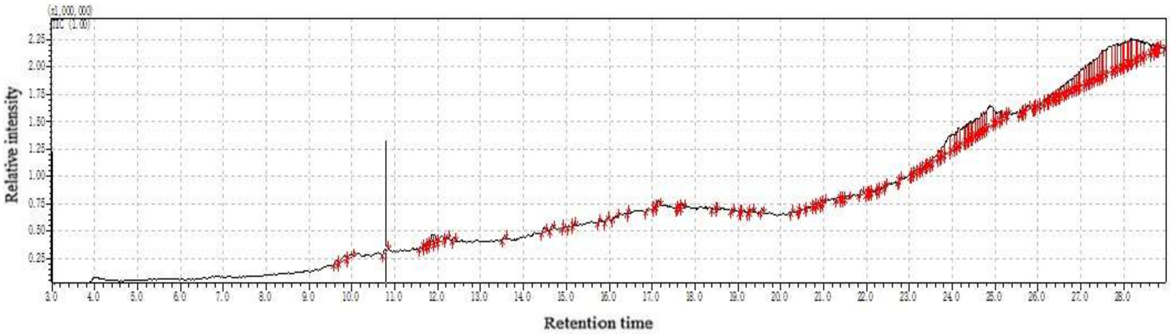

Supplementary Figure S2. GC-MS two-way data in chromatography for qingke flour

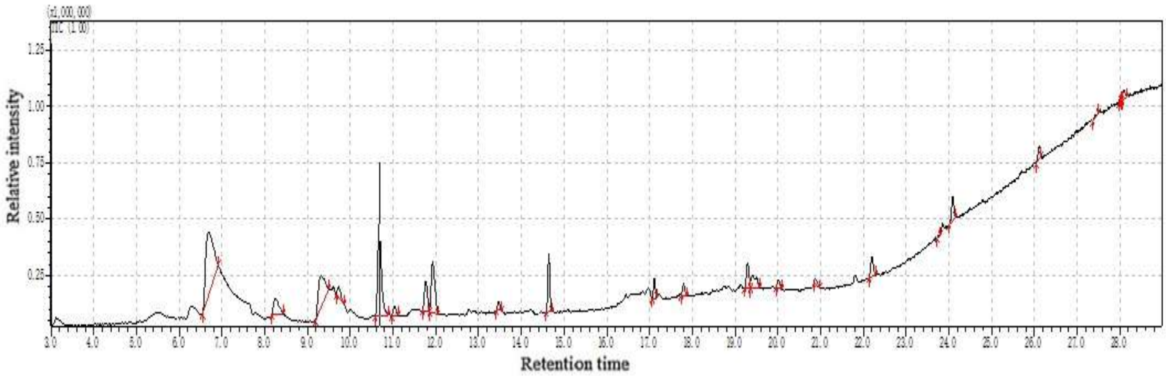

Supplementary Figure S3. GC-MS two-way data in chromatography for (90% qingke flour+10% Kudzu rootflour)

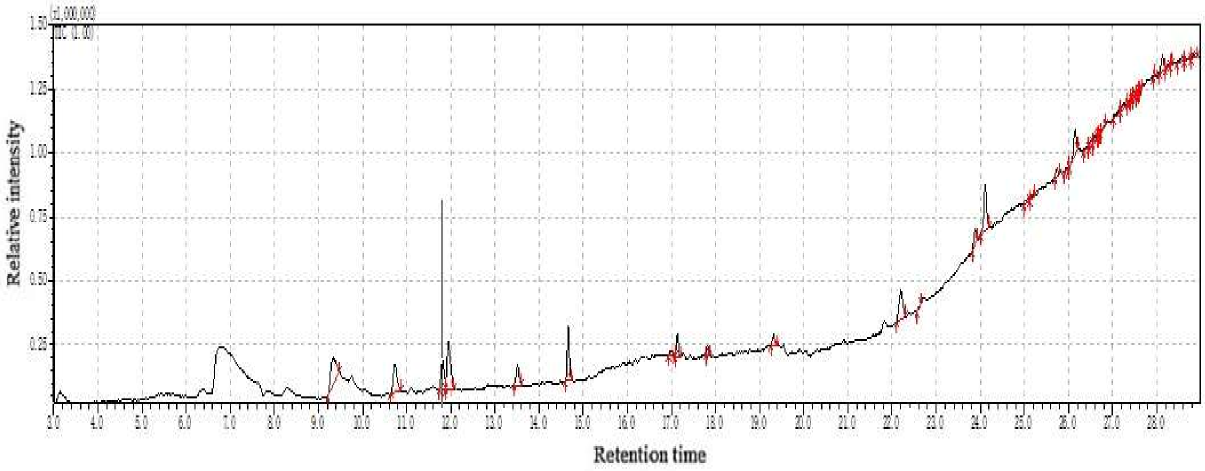

Supplementary Figure S4. GC-MS two-way data in chromatography for (90% highland barley flour +10% *Polygonatum sibiricum* Redouté flour)

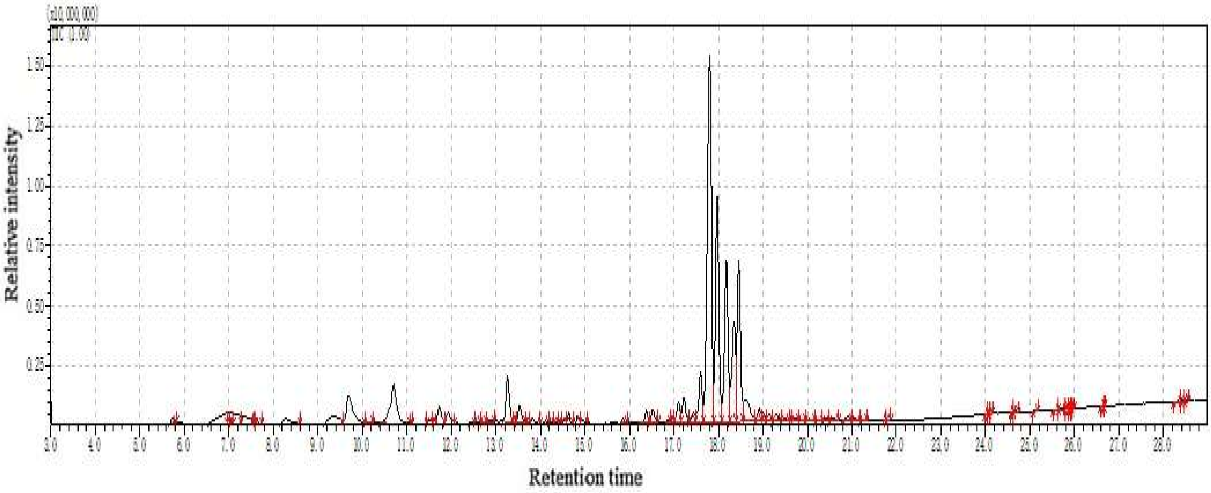

Supplementary Figure S5. GC-MS two-way data in chromatography for (90% qingke flour+10% dried ginger flour)
